# Flexible synthetic routes to poison-frog alkaloids of the 5,8-disubstituted indolizidine-class I: synthesis of common lactam chiral building blocks and application to the synthesis of (-)-**203A**, (-)-**205A**, and (-)-**219F**

**DOI:** 10.1186/1860-5397-3-29

**Published:** 2007-09-28

**Authors:** Naoki Toyooka, Dejun Zhou, Hideo Nemoto, H Martin Garraffo, Thomas F Spande, John W Daly

**Affiliations:** 1 Graduate School of Medicine and Pharmaceutical Sciences, University of Toyama, Sugitani 2630, Toyama, 930-0194, Japan; 2 Laboratory of Bioorganic Chemistry, National Institute of Diabetes and Digestive and Kidney Diseases, National Institutes of Health, DHHS, Bethesda, MD 20892, USA

## Abstract

**Background:**

The 5,8-disubstituted indolizidines are the largest class of poison-frog alkaloids found in anuran skin, and are of considerable interest because of their inhibitory effects on the neuronal nicotinic acetylcholine receptors. Many synthetic strategies for the construction of this nucleus have been reported: however, a flexible route has not been reported to date.

**Results:**

Synthesis of lactam chiral building blocks for the flexible synthesis of the title alkaloids has been achieved using a Michael-type conjugate addition reaction to a chiral cyclic enamine ester as the key step in constructing the trisubstituted piperidine ring system. To demonstrate the usefulness of these chiral building blocks, syntheses of (-)-**203A**, (-)-**205A** from **1**, and (-)-**219F** from **2** have been achieved.

**Conclusion:**

The total synthesis of (-)-**203A**, (-)-**205A**, and (-)-**219F** was achieved, and the absolute stereochemistry of natural **203A** was determined to be 5*S*, 8*R*, 9*S*. In addition, the relative stereochemistry of natural **219F** was determined.

## Introduction

**Figure 1:**
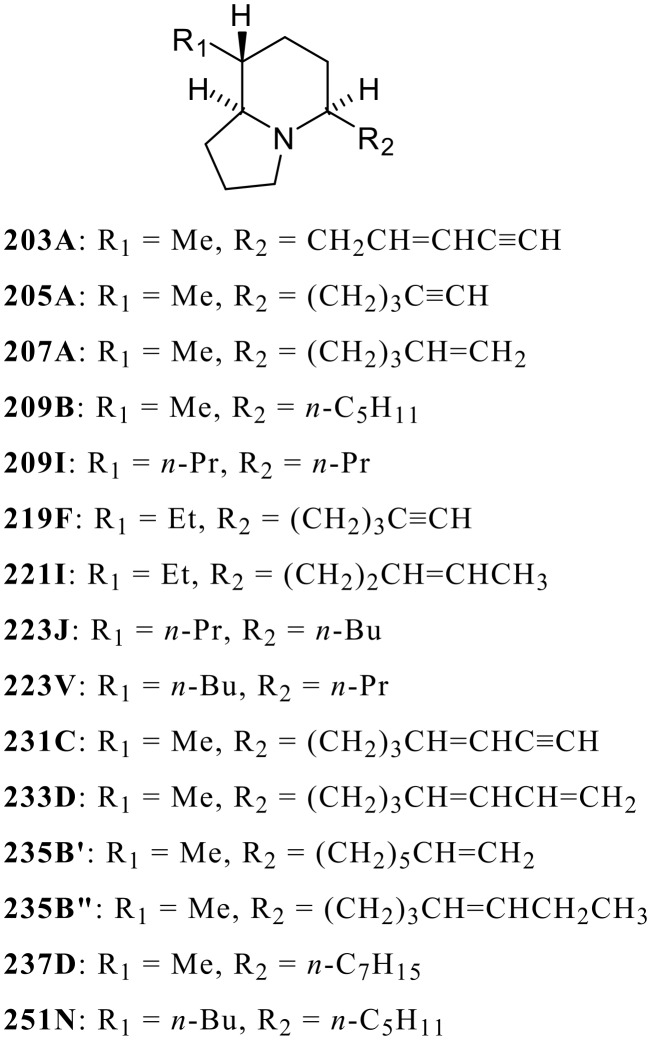
Representative examples of 5,8-Disubstituted Indolizidines.

The indolizidine ring system has been widely found in microbial, plant, and animal sources, and many natural products containing this ring system show interesting biological activities. [1] The skin extracts of poison-frogs are a rich source of indolizidines. [2] There are about 20 examples of 3,5-disubstituted indolizidines and about 80 of the 5,8-disubstituted indolizidines. Furthermore, many of such poison-frog alkaloids show significant activities, for example with nicotinic acetylcholine receptors (nAChRs) of the central nervous system. [3] Our syntheses and then biological evaluations of poison-frog alkaloids, [4–10] revealed that the 5,8-disubstituted indolizidine (-)-**235B'**, exhibited selective and potent blockade of α4β2-nAChRs. [11] Alkaloids of this class with various substituents at the 5- and 8-positions that have been synthesized are shown in Figure 1. All side-chain double bonds in these synthetic compounds have the *cis* (*Z*) configuration. Our flexible synthetic strategy provides a powerful tool for the synthesis of 5,8-disubstituted indolizidines, permitting detailed investigation of structure activity relationships for blockade of nAChRs by this class of alkaloids.

In this contribution, we describe the synthesis of the common lactam chiral building blocks that permit the flexible synthesis of 5,8-disubstituted indolizidines. Their application to the synthesis of (-)-**203A**, (-)-**205A**, and (-)-**219F** illustrates in detail the synthetic procedures employed. [12]

## Results and Discussion

**Figure 2:**
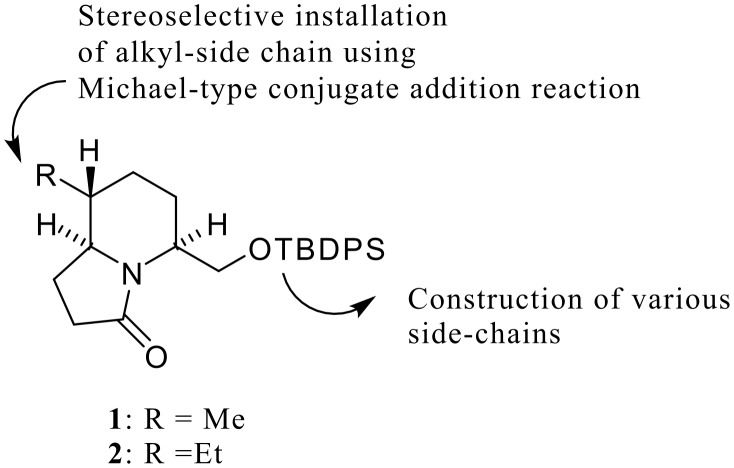
Synthetic Strategies to 5,8-Disubstituted Indolizidines from Chiral Building Blocks (**1**,**2**).

To realize the versatile synthesis of the 5,8-disubstituted indolizidine class of poison-frog alkaloids, we designed two lactam chiral building blocks (**1**, **2**). The substituent at the 8-position is stereoselectively created by our original Michael-type conjugate addition reaction. [13–14] Various substituents at the 5-position would be introduced using the protected hydroxymethyl side-chain (Figure 2).

The synthesis began with the known piperidone **3**, [15] which was treated with *n*-BuLi and then CbzCl to provide the Cbz-urethane **4**. Treatment of **4** with LiHMDS followed by 2-[*N*,*N*-bis(trifluoromethylsulfonyl)amino]-5-chloropyridine (Comins' reagent) [16] gave the enoltriflate **5** in good yield. The palladium-catalyzed carbon monoxide insertion reaction [17] in the presence of MeOH afforded the enaminoester **6**. The key Michael-type conjugate addition reaction of **6** with lithium dimethylcuprate or divinylcuprate proceeded smoothly to provide the trisubstituted piperidines (**7**, **8**) as single stereoisomers in excellent yields.

**Scheme 1:**
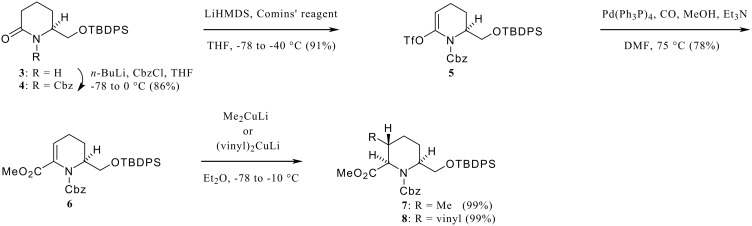
Construction of tri-substituted piperidine ring systems (**7**, **8**).

**Figure 3:**
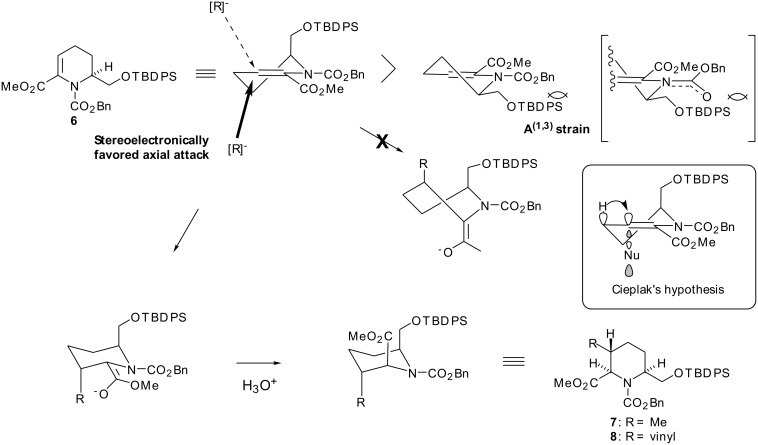
Stereochemical Course of Key Michael-type Conjugate Addition Reaction of **6**.

The stereochemical course of the above addition reaction can be rationalized as follows: The enamine ester **6** would adopt conformation **A** owing to A^(1,3)^ strain [18] between the benzyloxycarbonyl group on nitrogen and the substituent at the α-position, The methyl or vinyl anion then attacks from the α-orientation controlled by a stereoelectronic effect [19] producing the desired trisubstituted piperidine as a single isomer. This argument can also be explained by the Cieplak's hypothesis [20] as shown in Figure 3. Reduction of the ester moiety in **7** and **8** with Super-Hydride gave the corresponding alcohols (**9**, **10**) in good yield. Swern oxidation of **9** or **10** followed by Horner-Emmons reaction of the resulting aldehydes afforded the α,β-unsaturated esters (**11**, **12**) each in 97% yield. Hydrogenation of the double bond in **11** or **12** over 20% Pd(OH)_2_ and then treatment of the resulting deblocked amino alcohols with trimethylaluminum under Weinreb's conditions [21] gave rise to the lactams **1** and **2** in 71% and 68% overall yields, respectively.

**Scheme 2:**
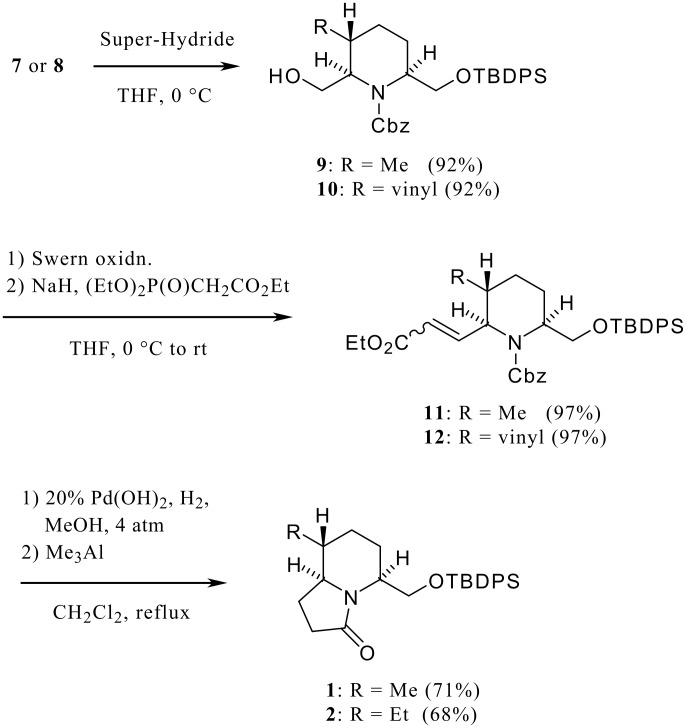
Synthesis of common lactam-type chiral building blocks (**1**, **2**).

**Scheme 3:**
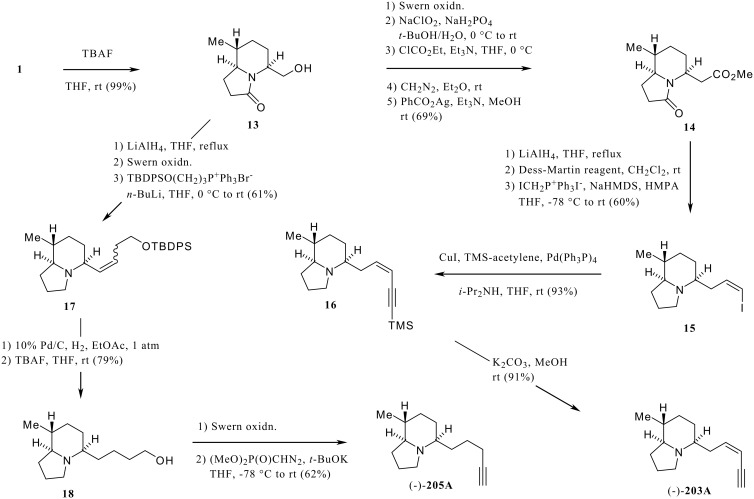
Synthesis of (-)-**203A** and (-)-**205A**.

**Scheme 4:**
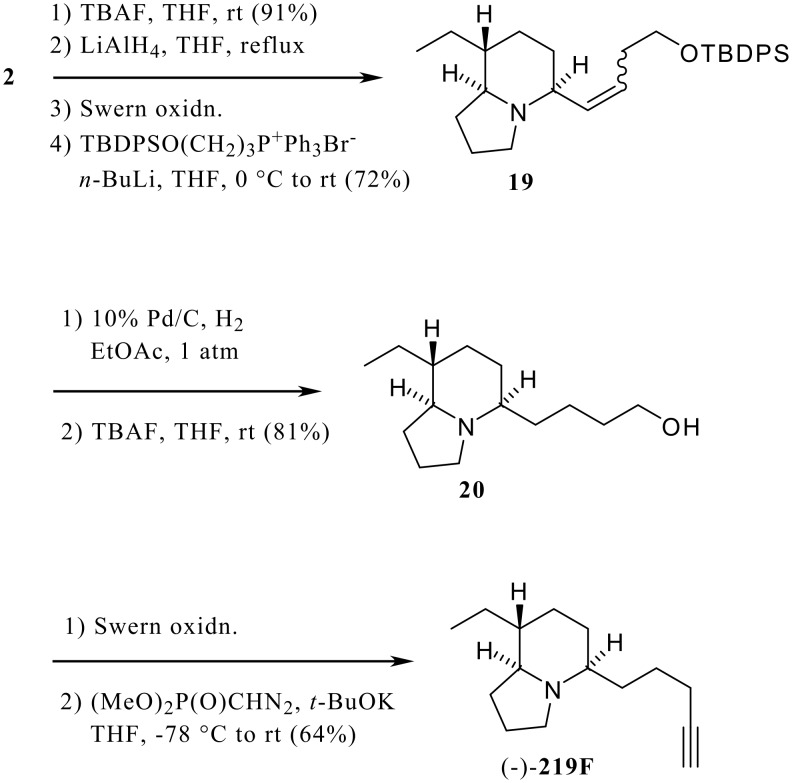
Synthesis of (-)-**219F**.

To demonstrate the utility of the chiral lactam building blocks, we conducted the total synthesis of indolizidines (-)-**203A** [22] and (-)-**205A** [23] from **1**, and (-)-**219F** [2] from **2**, respectively (Scheme 3, Scheme 4). Removal of the silyl protecting group in **1** was performed by treatment with TBAF to afford the corresponding alcohol **13**, which was converted to the homologated ester **14** via a two-step oxidation, followed by an Arndt-Eistert sequence of the resulting carboxylic acid. Reduction of both carbonyl groups in **14** with lithium aluminum hydride provided the alcohol, which was directly used for formation of *Z*-iodoolefin **15**. Thus, the Dess-Martin periodinane oxidation [24] of the alcohol, followed by Wittig reaction of the resulting aldehyde under Stork's reaction conditions, [25] gave the olefin. Purification by silica gel column chromatography afforded pure **15** in 60% isolated yield. The coupling reaction of **15** with TMS-acetylene under Sonogashira's conditions [26] gave rise to the product **16**. Finally, treatment of **16** with K_2_CO_3_ in MeOH provided (-)-**203A**. The GC-MS and GC-FTIR spectra of synthetic (-)-**203A** were identical with those of the natural product, and comparison of the optical rotation of the synthetic material ([α]_D_^26^ -94.5 (c 2.0, CHCl_3_) with the natural product, lit. [22] [α]_D_ -23.3 (c 0.3, CHCl_3_)) suggest that the absolute stereochemistry of natural **203A** is 5*S*, 8*R*, 9*S*.

We achieved the total synthesis of (-)-**205A** starting from **1** via **13** (Scheme 3). Lithium aluminum hydride reduction of **13** followed by Swern oxidation and Wittig reaction of the resulting aldehyde gave the olefin **17**. Hydrogenation of **17** over Pd/C, and treatment of the resulting indolizidine with TBAF provided the homologated alcohol **18**. Finally, the terminal triple bond was constructed by Seyferth-Gilbert reaction. [27] After oxidation of **18** under the Swern conditions, treatment of the resulting aldehyde with Seyferth-Gilbert reagent in the presence of *t*-BuOK furnished (-)-**205A**, whose spectral data were identical with reported values. [22,28]

In addition, (-)-**219F**, a 5,8-disubstituted indolizidine with an ethyl group at C-8, [2] was synthesized from **2** (Scheme 4). The lactam **2** was converted to the homologated alcohol **20** via **19** as used with **1** in the synthesis of (-)-**205A**, which was then transformed into (-)-**219F** using the Seyferth-Gilbert reaction after Swern oxidation of **20**.

Although the direct comparison of the NMR spectra of the synthetic alkaloid with the natural product was not possible due to the scarcity of natural product, the GC-MS and GC-FTIR spectra of the synthetic material were identical with those of natural product detected in the Madagascan mantellid frog, *Mantella betsileo*. Thus, the relative stereochemistry of natural **219F** was established.

In conclusion, we succeeded in the construction of chiral lactam building blocks (**1**, **2**) for the synthesis of three representative poison-frog alkaloids of the 5,8-disubstituted indolizidine class; these were alkaloids (-)-**203A**, (-)-**205A**, and (-)-**219F (**experimental details can be found in Supporting Information File 1). This flexible synthetic route starting from **1** or **2** will be amenable to any side-chain at the 5-position of these alkaloids. Such indolizidines are expected to show inhibitory effects on the nAChRs, and the biological results will be reported in due course.

## Supporting Information

File 1 Experimental details for the synthesis of (-)-**203A**, (-)-**205A**, and (-)-**219F**. Experimental data which includes experimental details on the spectral instruments, elemental analyzer.
