# Supplementary material for: Flexible synthetic routes to poison-frog alkaloids of the 5,8-disubstituted indolizidine-class I: synthesis of common lactam chiral building blocks and application to the synthesis of (-)-203A, (-)-205A, and (-)-219F
Source: Beilstein J Org Chem. 2007 Sep 28;3:29. doi: 10.1186/1860-5397-3-29 (PMC2147022; doi:10.1186/1860-5397-3-29)
Supplement: File 1 — Experimental details for the synthesis of (-)-203A, (-)-205A, and (-)-219F. Experimental data which includes experimental details on the spectral instruments, elemental analyzer. [file Beilstein_J_Org_Chem-03-29-s001.doc]

**Experimental details**

**General**

Melting points were determined with a Yanaco micro melting point apparatus and are uncorrected. ^1^H and ^13^C NMR spectra were taken on a Varian Gemini 300 or Unity Plus 500 spectrometer. ^1^H NMR spectra were recorded at the indicated field strength as solutions in CDCl_3_ unless otherwise indicated. Chemical shifts are given in parts per million (ppm, δ) downfield from TMS and are referenced to CHCl_3_ (7.26 ppm) as an internal standard. Splitting patterns are designated as s, singlet; d, doublet; t, triplet; q, quartet; m, multiplet; br, broad. ^13^C NMR spectra were recorded at the indicated field strength as solutions in CDCl_3_ unless otherwise indicated. Chemical shifts are given in ppm, (δ) downfield from TMS and are referenced to the center line of CDCl_3_ (77.0 ppm) as an internal standard. Carbon signals were assigned by a DEPT pulse sequence, q=methyl, t=methylene, d=methine, and s=quaternary carbons. Infrared spectra (IR) were measured with a Perkin-Elmer 1600 series FT-IR spectrophotometer. Mass spectra (MS) and high-resolution mass spectra (HRMS) were measured on a JEOL JMS-AX505HAD mass spectrometer. Optical rotations were measured on a JASCO DIP-1000 digital polarimeter. Column chromatography was performed with Kanto Kagaku silica gel 60N. GC-MS was performed with a Finnigan GCQ instrument fitted with a Restek Amine 5x column (30 cm x 0.25 mm) and a program 100 °C (1 min hold time) to 280 °C at 10 °C. GC-FTIR spectra were obtained with a narrow band HP model 5981 GC-FTIR infrared spectrophotometer interfaced with both an HP MSD, model 5971 and an HP 5890 gas chromatograph using the same temperature program as above and fitted with the same column except 0.32 mm i. d.

**Phenylmethyl (2*S*)-(-)-2-(*tert*-butyldiphenylsilyloxymethyl)-6-oxopiperidine-1-carboxylate (4)**

To a stirred solution of **3** (5.2 g, 14.2 mmol) in THF (30 mL) was added a solution of *n*-BuLi (1.6 M in hexane, 9.74 mL, 15.6 mmol) at –78 °C, and the resulting mixture was stirred at –78 °C for 30 min. To the reaction mixture was added ClCO_2_Bn (2.23 mL, 15.6 mmol) at the same temperature. The reaction mixture was stirred at –78 °C~0 °C for 3 h, and quenched with satd. NaHCO_3_ (aq). The aqueous mixture was extracted with CH_2_Cl_2_ (50 mL x 3). The organic extracts were combined, dried, and evaporated to give a pale yellow oil, which was chromatographed on silica gel (100 g, hexane : acetone = 20 : 1~15 : 1) to afford **4** (6.1 g, 86%) as a colorless oil.

IR (neat) 3069, 2956, 1772, 1716, 1255 cm^-1^; ^1^H NMR (500 MHz) δ 1.09 (9H, s), 1.73-1.78 (1H, m), 1.90-2.00 (2H, m), 2.15-2.19 (1H, m), 2.55 (2H, t, *J* = 7.0 Hz), 3.77 (2H, d-like, *J* = 5.5 Hz), 4.45-4.49 (1H, m), 5.24 (2H, ABq, *J* = 12.0 Hz), 7.35-7.48 (11H, br m), 7.66-7.70 (4H, m); ^13^C NMR (75 MHz) δ 17.61 (t), 19.13 (s), 24.42 (t), 26.78 (q), 34.87 (t), 56.26 (d), 64.22 (t), 68.25 (t), 127.56 (d), 127.72 & 127.95 (each d), 128.31 (d), 129.60 (d), 129.63 (s), 132.54 & 132.76 (each s), 135.18 (s), 135.31 & 135.38 (each d), 153.80 (s), 171.60 (s); MS: 444 (M^+^-57); HRMS: Calcd for C_26_H_26_NO_4_Si 444.1629; Found 444.1643; [α]_D_^26^ –52.3 (*c* 1.25, CHCl_3_).

**Phenylmethyl (2*S*)-(-)-2-(*tert*-butyldiphenylsilyloxymethyl)-6-trifluoromethanesulfonyloxy-**

**3,4-dihydro-2*H*-pyridine-1-carboxylate (5)**

To a stirred solution of **4** (6.1 g, 12.2 mmol) in THF (15 mL) was added a solution of LiHMDS, prepared from 1,1,1,3,3,3-hexamethyldisilazane (3.1 mL, 14.6 mmol) and *n*-BuLi (1.6 M in hexane, 9.2 mL, 14.6 mmol) in THF (12 mL) at 0 °C for 30 min., at –78 °C, and the reaction mixture was stirred at the same temperature for 30 min. To the reaction mixture was added dropwise a solution of 2-[*N*,*N*-bis(trifluoromethylsulfonyl)amino]-5-chloropyridine (Comins’ reagent) (5.25 g, 13.4 mmol) in THF (6 mL) via a double-tipped stainless steel needle at –78 °C, and the resulting mixture was stirred at –78 °C~-40 °C for 30 min. The reaction was quenched with satd. NH_4_Cl (aq), and the aqueous mixture was extracted with Et_2_O (50 mL x 3). The ethereal layers were combined, dried, and evaporated to give a pale yellow oil, which was chromatographed on silica gel (100 g, hexane : acetone = 60 : 1~50 : 1) to afford **5** (7.0 g, 91%) as colorless oil.

IR (neat) 3071, 2958, 1730, 1683, 1215 cm^-1^; ^1^H NMR (500 MHz) δ 1.12 (9H, s), 1.73-1.80 (1H, m), 1.96-2.08 (2H, br m), 2.17-2.23 (1H, br m), 3.62-3.67 (1H, m), 3.85-3.92 (1H, m), 4.75-4.80 (1H, m), 5.22-5.42 (3H, m), 7.38-7.49 (11H, br m), 7.67-7.74 (4H, m); ^13^C NMR (75 MHz) δ 19.03 (s), 19.24 (t), 22.22 (t), 26.76 (q), 55.77 (d), 60.80 (t), 68.47 (t), 106.41 (d), 126.06 (d), 127.58 & 128.16 (each d), 128.22 & 128.35 (each d), 129.63 (d), 133.03 & 133.08 (each s), 135.15 (s), 135.34 & 135.38 (each d), 135.64 (s), 138.00 & 139.16 (each s), 149.13 (d), 153.17 (s); MS: 576 (M^+^-57); HRMS: Calcd for C_27_H_25_F_3_NO_6_SSi 576.1122; Found 576.1127; [α]_D_^26^ –57.2 (*c* 1.26, CHCl_3_).

**Phenylmethyl 2-methyl (2*S*)-(-)-6-(*tert*-butyldiphenylsilyloxymethyl)-5,6-dihydro-**

**4*H*-pyridine-1,2-dicarboxylate (6)**

To a stirred solution of **5** (7 g, 11.1 mmol) in DMF (45 mL) was added Pd(Ph_3_P)_4_ (640 mg, 0.55 mmol), and the resulting mixture was stirred at room temperature under CO balloon pressure for 30 min. To the mixture were added Et_3_N (6.3 mL, 45.3 mmol) and MeOH (18 mL, 445.3 mmol), and then the reaction mixture was stirred at 75 °C under CO balloon pressure for 15 h. After cooling, the reaction mixture was diluted with H_2_O (100 mL) and brine (25 mL), and the aqueous mixture was extracted with Et_2_O (50 mL x 4). The organic layers were combined, dried, and evaporated to give pale yellow oil, which was chromatographed on silica gel (80 g, hexane : acetone = 50 : 1~30 : 1) to afford **6** (4.7 g, 78%) as a pale yellow oil.

IR (neat) 3069, 2955, 1737, 1714, 1269 cm^-1^; ^1^H NMR (500 MHz) δ 1.09 (9H, s), 1.82-1.89 (1H, m), 1.95-2.03 (1H, m), 2.06-2.15 (1H, m), 2.17-2.19 (1H, m), 3.48 (3H, br), 3.57 (1H, t-like, J = 6.5 Hz), 3.81-3.84 (1H, m), 4.68 (1H, br), 5.08 & 5.26 (2H, ABq, J = 11.9 Hz), 6.01 (1H, t-like, J = 3.8 Hz), 7.33-7.49 (11H, br m), 7.65-7.73 (4H, m); ^13^C NMR (75 MHz) δ 19.24 (t), 19.48 (s), 22.44 (t), 26.76 (q), 51.73 (q), 52.60 (d), 61.24 (t), 67.89 (t), 126.75 (d), 127.51 (d), 128.00 (d), 128.29 (d), 129.54 (d), 130.39 (s), 133.11 & 133.26 (each s), 135.38 (d), 135.64 (s), 153.79 (s), 165.21 (s); MS: 543 (M^+^); HRMS: Calcd for C_32_H_37_NO_5_Si 543.2442; Found 543.2465; [α]_D_^26^ –37.4 (*c* 1.30, CHCl_3_).

**Phenylmethyl 2-methyl (2*R*,3*R*,6*S*)-(-)-6-(*tert*-butyldiphenylsilyloxymethyl)-**

**3-methylpiperidine-1,2-dicarboxylate (7)**

To a stirred suspension of CuI (8.2 g, 43.1 mmol) in Et_2_O (50 mL) was added a solution of MeLi (0.98 M in Et_2_O, 88 mL, 87 mmol) at –78 °C, and the resulting suspension was stirred at –78 to –35 °C for 20 min. The resulting solution was cooled to –78 °C, and a solution of **6** (4.7 g, 8.7 mmol) in Et_2_O (15 mL) was added dropwise via a double-tipped stainless steel needle to the above reaction mixture at –78 °C. The temperature was gradually raised to –10 °C, and then the reaction was quenched with satd. NH_4_Cl (aq). The aqueous mixture was diluted with CH_2_Cl_2_, and the insoluble material was removed through a celite pad. The filtrate was separated and the aqueous layer was extracted with CH_2_Cl_2_.All the organic layers were combined, dried, and evaporated to give a pale yellow oil, which was chromatographed on silica gel (70 g, hexane : acetone = 50 : 1~30 : 1) to afford **7** (4.8 g, 99%) as a colorless oil.

IR (neat) 3070, 2954, 1746, 1703 cm^-1^; ^1^H NMR (500 MHz) δ 1.09 (9H, s), 1.10 (3H, d, *J* = 7.4 Hz), 1.22-1.28 (1H, m), 1.57-1.62 (1H, m), 1.88-1.92 (2H, m), 2.49 (1H, br), 3.42 (3H, s), 3.52 (1H, t-like, *J* = 10.4 Hz), 3.79 (1H, dd, *J* = 10.4, 4.6 Hz), 4.43 (1H, br), 4.52 (1H, br), 5.14 & 5.21 (2H, ABq, *J* = 12.6 Hz), 7.26-7.47 (11H, br m), 7.66-7.70 (4H, m); ^13^C NMR (75 MHz) δ 18.16 (q), 18.25 (t), 19.30 (s), 21.97 (t), 26.88 (q), 28.06 (d), 51.74 (q), 52.18 (d), 58.62 (d), 62.41 (t), 67.24 (t), 127.38 & 127.45 (each d), 127.67 (d), 128.24 (d), 129.41 & 129.44 (each d), 130.12 (d), 133.37 & 133.47 (each s), 135.33 & 135.36 (each d), 136.46 (s), 156.47 (s), 172.49 (s); MS: 502 (M^+^-57); HRMS: Calcd for C_29_H_32_NO_5_Si 502.2048; Found 502.2039; [α]_D_^26^ -0.43 (*c* 2.73, CHCl_3_).

**Phenylmethyl 2-methyl (2*R*,3*S*,6*S*)-(-)-6-(*tert*-butyldiphenylsilyloxymethyl)-3-vinylpiperidine-**

**1,2-dicarboxylate (8)**

To a stirred solution of tetravinyltin (4 mL, 24.5 mmol) in Et_2_O (10 mL) was added a solution of MeLi (0.98 M in Et_2_O, 100 mL, 98 mmol) at 0 °C, and the reaction mixture was stirred at 0 °C for 30 min. To a stirred suspension of CuI (9.33 g, 49.0 mmol) in Et_2_O (20 mL) was added the solution of vinyllithium prepared above at –78 °C, and the resulting mixture was stirred at –78 °C~-35°C for 30 min. To the reaction mixture was added dropwise a solution of **6** (6.1 g, 11.2 mmol) in Et_2_O (20 mL) via a double-tipped stainless steel needle at –78 °C. The reaction mixture was stirred at-78 °C~-10 °C for 1 h, and then the reaction was quenched with satd. NH_4_Cl (aq). The aqueous mixture was diluted with CH_2_Cl_2_, and the insoluble material was filtered off. The organic layer was separated and the aqueous layer was extracted with CH_2_Cl_2_ (30 mL x 2). The organic layer and extracts were combined, dried, and evaporated to give a pale yellow oil, which was chromatographed on silica gel (100 g, hexane : acetone = 25 : 1~20 : 1) to afford **8** (6.3 g, 99%) as a pale yellow oil.

IR (neat) 3074, 2953, 1744, 1698, 1269, 1113 cm^-1^; ^1^H NMR (500 MHz) δ 1.05 (9H, s), 1.21 (1H, br), 1.44 (1H, br), 1.80 (1H, m), 1.87 (1H, br), 3.02 (1H, br), 3.40 (3H, s), 3.47 (1H, t-like, *J* = 9.9 Hz), 3.74 (1H, m), 4.40 (1H, br), 4.82 (1H, br), 5.11-5.19 (4H, m), 5.82-5.89 (1H, m), 7.28-7.45 (11H, br m), 7.64 (4H, d-like, *J* = 6.4 Hz); ^13^C NMR (75 MHz) δ 18.43 (t), 19.26 (s), 20.70 (t), 26.84 (q), 36.66 (d), 51.84 (q), 52.13 (d), 55.81 (d), 61.97 (t), 67.26 (t), 115.28 (t), 127.37 & 127.45 (each d), 127.67 (d), 128.06 (d), 128.21 (d), 129.41 & 129.44 (each d), 133.26 & 133.37 (each s), 135.30 & 135.33 (each d), 136.38 (s), 138.55 (d), 156.10 (s), 172.17 (s); MS: 514 (M^+^-57); HRMS: Calcd for C_30_H_32_NO_5_Si 514.2048; Found 514.2067; [α]_D_^26^ –8.80 (*c* 1.69, CHCl_3_).

**Phenylmethyl (2*R*,3*R*,6*S*)-(+)-6-(*tert*-butyldiphenylsilyloxymethyl)-**

**2-hydroxymethyl-3-methylpiperidine-1-carboxylate (9)**

To a stirred solution of **7** (4.8 g, 8.6 mmol) in THF (30 mL) was added a solution of lithium triethylborohydride (Super-Hydride®, 1 M in THF, 18.9 mL, 18.9 mmol) at 0 °C, and the reaction mixture was stirred at 0 °C for 2h. The reaction was quenched with small pieces of ice, and the mixture was diluted with CH_2_Cl_2_. The organic layer was dried and evaporated to give a colorless oil, which was chromatographed on silica gel (80 g, hexane : acetone = 10 : 1~8 : 1) to afford **9** (4.2 g, 92%) as a colorless oil.

IR (neat) 3445, 3070, 2957, 1697, 1112 cm^-1^; ^1^H NMR (500 MHz) δ 1.06-1.07 (12H, br s-like), 1.15-1.22 (1H, m), 1.46 (1H, br), 1.65 (1H, br), 1.85 (2H, br), 2.92 (1H, br), 3.54-3.75 (4H, br m), 4.10 (1H, br), 4.50 (1H, br), 5.10 (1H, br), 5.21 (1H, d-like, *J* = 12.4 Hz), 7.34-7.47 (11H, br m), 7.69 (4H, br); ^13^C NMR (75 MHz) δ 19.17 (q), 19.37 (s), 19.82 (t), 22.73 (t), 26.80 (q), 27.52 (d), 51.03 (d), 59.07 (d), 65.03 (t), 67.29 (t), 126.75 (d), 127.58 & 127.61 (each d), 127.71 (d), 128.31 (d), 129.62 (d), 132.85 (s), 135.33 & 135.39 (each d), 136.53 (s), 157.57 (s); MS: 474 (M^+^-57); HRMS: Calcd for C_28_H_32_NO_4_Si 474.2099; Found 474.2093; [α]_D_^26^ +8.80 (*c* 1.59, CHCl_3_).

**Phenylmethyl (2*R*,3*S*,6*S*)-(+)-6-(*tert*-butyldiphenylsilyloxymethyl)-**

**2-hydroxymethyl-3-vinylpiperidine-1-carboxylate (10)**

To a stirred solution of **8** (6.04 g, 10.58 mmol) in THF (80 mL) was added a solution of Super-Hydride® (1 M in THF, 23 mL, 23.0 mmol) at 0 °C, and the resulting solution was stirred at 0 °C for 2 h. The reaction was quenched with small pieces of ice, and the mixture was diluted with CH_2_Cl_2_. The organic layer was dried, and evaporated to give a colorless oil, which was chromatographed on silica gel (70 g, hexane : acetone = 20 : 1~8 : 1) to afford **10** (5.3 g, 92%) as a colorless oil.

IR (neat) 3445, 3070, 2932, 2858, 1695, 1112 cm^-1^; ^1^H NMR (500 MHz) δ 1.09 (9H, s), 1.30-1.43 (1H, m), 1.47 (1H, br), 1.67-1.72 (1H, m), 1.84-1.90 (1H, m), 2.43 (1H, br), 3.14 (1H, br), 3.58-3.75 (4H, br m), 4.36 (1H, br m), 4.51 (1H, br), 5.09-5.24 (4H, m), 5.89 (1H, m), 7.33-7.48 (11H, m), 7.70-7.71 (4H, br); ^13^C NMR (75 MHz) δ 19.14 (s), 20.15 (t), 22.67 (t), 26.78 (q), 36.79 (d), 50.98 (d), 56.36 (d), 64.41 (t), 67.33 (t), 115.04 (t), 127.58 (d), 127.72 (d), 128.29 (d), 129.62 (d), 132.79 (s), 135.31 (d), 135.36 (d), 136.41 (s), 140.02 (d), 157.15 (s); MS: 486 (M^+^-57); HRMS: Calcd for C_29_H_32_NO_4_Si 486.2099; Found 486.2107; [α]_D_^26^ +14.2 (*c* 1.11, CHCl_3_).

**Phenylmethyl (2*R*,3*R*,6*S*)-6-(*tert*-butyldiphenylsilyloxymethyl)-2-(2-ethoxycarbonylvinyl)-**

**3-methylpiperidine-1-carboxylate (11)**

To a stirred solution of (COCl)_2_ (1.04 mL, 11.86 mmol) in CH_2_Cl_2_ (15 mL) was added DMSO (1.68 mL, 23.73 mmol) at –78 °C, and the resulting mixture was stirred at the same temperature for 5 min. To the reaction mixture was added dropwise a solution of **9** (4.2 g, 7.91 mmol) in CH_2_Cl_2_ (6 mL) via a double-tipped stainless steel needle at –78 °C. The resulting solution was stirred at –78 °C for 30 min. and then triethylamine (4.92 mL, 35.59 mmol) was added to the reaction mixture. The reaction mixture was warmed to 0 °C for 1 h., and quenched with H_2_O. The aqueous mixture was extracted with Et_2_O (50 mL x 3), and the organic extracts were combined, dried, and evaporated to give a pale yellow oil, which was used directly in the next step.

To a stirred suspension of NaH (60%, 350 mg, 8.7 mmol) in THF (15 mL) was added (EtO)_2_P(O)CH_2_CO_2_Et (1.74 mL, 8.7 mmol) at 0 °C, and the reaction mixture was stirred at 0 °C for 30 min. To the resulting mixture was added dropwise a solution of the above aldehyde in THF (6 mL) via a double-tipped stainless steel needle at 0 °C. The reaction mixture was stirred at room temperature for 20 h. The reaction was quenched with H_2_O, and the aqueous mixture was extracted with CH_2_Cl_2_ (40 mL x 4). The organic extracts were combined, dried, and evaporated to give a pale yellow oil, which was chromatographed on silica gel (80 g, hexane : acetone = 20 : 1~10 : 1) to afford **11** (4.6 g, 97%) as a 2 : 1 mixture of the *E*- and *Z*-isomers as a pale yellow oil.

^1^H NMR (500 MHz) δ 1.03-1.07 (12H, br s-like), 1.21 (3H, t, *J* = 7.1 Hz), 1.50-1.66 (3H, br m), 1.80-1.94 (2H, br m), 3.44-3.68 (2H, br m), 4.05-4.18 (2H, m), 4.48-4.54 (2H, br m), 5.01-5.20 (2H, m), 5.62 & 5.87 (1H, m), 6.22 & 6.89 (1H, m), 7.28-7.43 (11H, br m), 7.64 (4H, t-like, *J* = 7.4 Hz).

**Phenylmethyl (2*R*,3*S*,6*S*)-6-(*tert*-butyldiphenylsilyloxymethyl)-2-(2-ethoxycarbonylvinyl)-**

**3-vinyl-piperidine-1-carboxylate (12)**

To a stirred solution of (COCl)_2_ (1.28 mL, 14.64 mmol) in CH_2_Cl_2_ (20 mL) was added DMSO (2.1 mL, 29.28 mmol) at –78 °C, and the resulting mixture was stirred at the same temperature for 5 min. To the reaction mixture was added dropwise a solution of **10** (5.3 g, 9.76 mmol) in CH_2_Cl_2_ (9 mL) via a double-tipped stainless steel needle at –78 °C. The resulting solution was stirred at –78 °C for 30 min. and then triethylamine (6.1 mL, 43.92 mmol) was added to the reaction mixture. The reaction mixture was warmed to 0 °C for 1 h., and quenched with H_2_O. The aqueous mixture was extracted with Et_2_O (50 mL x 3), and the organic extracts were combined, dried, and evaporated to give a pale yellow oil, which was used directly in the next step.

To a stirred suspension of NaH (60%, 430 mg, 10.74 mmol) in THF (30 mL) was added (EtO)_2_P(O)CH_2_CO_2_Et (2.2 mL, 10.74 mmol) at 0 °C, and the reaction mixture was stirred at 0 °C for 30 min. To the resulting mixture was added dropwise a solution of the above aldehyde in THF (9 mL) via a double-tipped stainless steel needle at 0 °C. The reaction mixture was stirred at room temperature for 22 h. The reaction was quenched with H_2_O, and the aqueous mixture was extracted with CH_2_Cl_2_ (50 mL x 4). The organic extracts were combined, dried, and evaporated to give a pale yellow oil, which was chromatographed on silica gel (80 g, hexane : acetone = 20 : 1~10 : 1) to afford **12** (5.78 g, 97%) as a 2 : 1 mixture of the *E*- and *Z*-isomers as a pale yellow oil.

**(5*S*,8*R*,9*S*)-(-)-5-(*tert*-Butyldiphenylsilyloxymethyl)-8-methylhexahydroindolizin-3-one (1)**

To a stirred solution of **11** (4.6 g, 7.68 mmol) in MeOH (20 mL) was added 20% Pd(OH)_2_/C (500 mg), and the resulting suspension was hydrogenated at 4 atm under hydrogen atmosphere for 44 h. The catalyst was filtered off and the filtrate was evaporated to give a pale yellow oil. To the solution of this oil in CH_2_Cl_2_ (50 mL) was added a solution of Me_3_Al (1 M in hexane, 9.2 mL, 9.2 mmol) at 0 °C, and the resulting solution was refluxed for 15 h. After cooling, the reaction was quenched with 10% HCl (aq), and the organic layer was separated. The aqueous layer was extracted with CH_2_Cl_2_ (30 mL x 3), and the organic layer and extracts were combined, dried, and evaporated to give a pale yellow oil, which was chromatographed on silica gel (60 g, hexane : acetone = 20 : 1~10 : 1) to afford **1** (2.3 g, 71%) as a colorless solid (mp 78~80 °C).

IR (KBr) 3066, 2958, 1752, 1688, 1109, 1088 cm^-1^; ^1^H NMR (500 MHz) δ 0.91 (3H, d, J = 6.4 Hz), 1.12 (9H, s), 1.13-1.21 (1H, m), 1.29-1.35 (1H, m), 1.52-1.59 (2H, m), 1.85-1.89 (1H, m), 2.11-2.16 (2H, m), 2.25-2.28 (2H, m), 2.95 (1H, dt, *J* = 9.4, 7.3 Hz), 3.34-3.38 (1H, m), 4.20 (1H, t, *J* =9.0 Hz), 4.62 (1H, dd, *J* = 9.0, 4.2 Hz), 7.38-7.45 (6H, m), 7.72 –7.74 (4H, br); ^13^C NMR (75 MHz) δ 17.61 (q), 19.27 (s), 24.09 (t), 26.89 (q), 27.59 (t), 31.20 (t), 31.34 (t), 37.26 (d), 57.55 (d), 63.67 (t), 64.36 (d), 127.35 & 129.29 (each d), 133.53 & 133.58 (each d), 135.31 (s), 135.36 (d), 174.37 (s); MS: 364 (M^+^-57); HRMS: Calcd for C_22_H_26_NO_2_Si 364.1731; Found 364.1755; [α]_D_^26^ –62.5 (*c* 1.74, CHCl_3_).

**(5*S*,8*R*,9*S*)-(-)-5-(*tert*-Butyldiphenylsilyloxymethyl)-8-ethylhexahydroindolizin-3-one (2)**

To a stirred solution of **12** (1.6 g, 2.61 mmol) in MeOH (10 mL) was added 20% Pd(OH)_2_/C (100 mg), and the resulting suspension was hydrogenated at 4 atm under hydrogen atmosphere for 44 h. The catalyst was filtered off and the filtrate was evaporated to give a pale yellow oil. To the solution of this oil in CH_2_Cl_2_ (30 mL) was added a solution of Me_3_Al (1 M in hexane, 3.1 mL, 3.1 mmol) at 0 °C, and the resulting solution was refluxed for 17 h. After cooling, the reaction was quenched with 10% HCl (aq), and the organic layer was separated. The aqueous layer was extracted with CH_2_Cl_2_ (30 mL x 3), and the organic layer and extracts were combined, dried, and evaporated to give a pale yellow oil, which was chromatographed on silica gel (50 g, hexane : acetone = 20 : 1~10 : 1) to afford **2** (771 mg, 68%) as a colorless solid (mp 84~87 °C).

IR (KBr) 3070, 2931, 1690, 1112 cm^-1^; ^1^H NMR (500 MHz) δ 0.88 (3H, t, *J* = 7.3 Hz), 0.90-1.18 (3H, m), 1.11 (9H, s), 1.44-1.60 (3H, br m), 1.87-1.91 (1H, m), 2.04-2.11 (2H, m), 2.19-2.22 (2H, m), 3.00 (1H, dt, *J* = 9.4, 7.3 Hz), 3.40-3.43 (1H, m), 4.11 (1H, t, *J* = 9.0 Hz), 4.56 (1H, dd, *J* = 9.0, 4.2 Hz), 7.35-7.40 (6H, m), 7.71-7.74 (4H, m); ^13^C NMR (75 MHz) δ 10.44 (q), 18.92 (s), 24.22 (t), 24.26 (t), 26.31 (t), 26.59 (q), 30.89 (t), 43.10 (d), 56.36 (d), 61.94 (d), 63.22 (t), 127.04 (d), 128.99 (d), 133.13 (d), 134.96 & 135.00 (each s), 173.96 (s); MS: 378 (M^+^-57); HRMS: Calcd for C_23_H_28_NO_2_Si 378.1888; Found 378.1897; [α]_D_^26^ –63.1 (*c* 1.71, CHCl_3_).

**(5*S*,8*R*,9*S*)-(-)-5-Hydroxymethyl-8-methylhexahydroindolizin-3-one (13)**

To a stirred solution of **1** (300 mg, 0.71 mmol) in THF (5 mL) was added a solution of TBAF (1M in THF, 0.78 mL, 0.78 mmol) at 0 °C, and the resulting mixture was stirred at room temperature for 13 h. The reaction mixture was evaporated, and the residue was chromatographed on silica gel (10 g, hexane : acetone = 20 : 1~2 : 1) to afford **13** (129 mg, 99%) as a colorless oil.

IR (neat) 3342, 2929, 1663 cm^-1^; ^1^H NMR (500 MHz) δ 0.86 (3H, d, *J* = 6.5 Hz), 1.09-1.25 (2H, br m), 1.28-1.37 (1H, m), 1.53-1.63 (2H, m), 1.76-1.80 (1H, m), 2.15-2.21 (1H, m), 2.30-2.37 (2H, m), 2.92-2.97 (1H, m), 3.06 (1H, br), 3.72 (1H, d-like, *J* = 12.9 Hz), 3.81-3.86 (1H, m), 4.77 (1H, br); ^13^C NMR (75 MHz) δ 17.28 (q), 23.90 (t), 28.12 (t), 31.44 (t), 32.30 (t), 38.10 (d), 60.48 (d), 63.70 (t), 65.84 (d), 175.39 (s); MS: 183 (M^+^); HRMS: Calcd for C_10_H_17_NO_2_ 183.1258; Found 183.1269; [α]_D_^26^ –53.6 (*c* 1.25, CHCl_3_).

**Methyl (5*S*,8*R*,9*S*)-(-)-(8-methyl-3-oxooctahydroindolizin-5-yl)acetate (14)**

To a stirred solution of (COCl)_2_ (0.15 mL, 1.73 mmol) in CH_2_Cl_2_ (3 mL) was added DMSO (0.25 mL, 3.45 mmol) at –78 °C, and the resulting mixture was stirred at the same temperature for 5 min. To the reaction mixture was added dropwise a solution of **13** (211 mg, 1.15 mmol) in CH_2_Cl_2_ (2 mL) via a double-tipped stainless steel needle at –78 °C, and the reaction mixture was stirred at –78 °C for 30 min. Triethylamine (0.72 mL, 5.18 mmol) was added to the reaction mixture, and the reaction mixture was warmed to 0 °C for 1 h. The reaction was quenched with H_2_O, and the aqueous mixture was extracted with Et_2_O (15 mL x 3). The organic layers were combined, dried, and evaporated to give a pale yellow oil, which was used directly in the next step.

To a stirred solution of the above aldehyde in *t*-BuOH (6 mL) were added 2-methyl-2-butene (4.9 mL, 46.2 mmol), NaH_2_PO_4_•2H_2_O (1.8 g, 11.5 mmol), and then a solution of NaClO_2_ (80%, 783 mg, 6.9 mmol) in H_2_O (2 mL) at 0 °C. The reaction mixture was stirred at room temperature for 1 h. and quenched with satd. NaHSO_3_ (aq) at 0 °C. The aqueous mixture was acidified with 10% HCl (aq), saturated with solid NaCl, and extracted with EtOAc (10 mL x 10). The organic extracts were conbined, dried, and evaporated to give a pale yellow oil, which was used directly in the next step.

To a stirred solution of the above carboxylic acid in THF (7 mL) were added Et_3_N (0.18 mL, 1.27 mmol) and ClCO_2_Et (0.12 mL, 1.27 mmol) at 0 °C, and the resulting suspension was stirred at the same temperature for 1 h. The insoluble material was removed by filtration, and the filtrate was evaporated to give a pale yellow oil, which was used directly in the next step.

To a stirred solution of the above oil in Et_2_O (15 mL) was added a solution of CH_2_N_2_ in Et_2_O at 0 °C, and the yellow solution was stirred at room temperature for 12 h. The solvent was removed in vacuo and the residue was dissolved in MeOH. To the MeOH solution were added PhCO_2_Ag (26 mg, 0.11 mmol) and Et_3_N (0.32 mL, 2.31 mmol), and the resulting suspension was stirred at room temperature in the dark for 18 h. The insoluble material was removed by filtration, and the filtrate was evaporated to give a pale brown oil, which was chromatographed on silica gel (20 g, hexane : acetone = 20 : 1~10 : 1) to afford **14** (179 mg, 69%) as a pale yellow oil.

IR (neat) 2931, 1738, 1672 cm^-1^; ^1^H NMR (500 MHz) δ 0.91 (3H, d, J = 6.4 Hz), 1.16-1.32 (3H, br m), 1.40-1.48 (1H, m), 1.53-1.61 (1H, m), 1.63-1.67 (1H, m), 1.79-1.84 (1H, m), 2.18-2.20 (1H, m), 2.30-2.34 (2H, m), 2.48 (1H, dd, J = 16.6, 4.7 Hz), 2.99 (1H, q-like, J = 9.4 Hz), 3.49 (1H, dd, J = 16.6, 8.5 Hz), 3.61 (1H, br), 3.71 (3H, s); ^13^C NMR (75 MHz) δ 17.28 (q), 23.69 (t), 31.39 (t), 32.07 (t), 32.33 (t), 37.77 (t), 37.88 (d), 51.34 (q), 52.68 (d), 65.16 (d), 171.93 (s), 174.98 (s); MS: 225 (M^+^); HRMS: Calcd for C_12_H_19_NO_3_ 225.1364; Found 225.1349; [α]_D_^26^ –70.7 (*c* 1.97, CHCl_3_).

**(5*S*,8*R*,9*S*)-(-)-5-(3-Iodoallyl)-8-methyloctahydroindolizine (15)**

To a stirred solution of **14** (112 mg, 0.50 mmol) in THF (10 mL) was added LiAlH_4_ (56 mg, 1.49 mmol) at 0 °C, and the resulting suspension was refluxed for 16 h. After cooling, the reaction was quenched with 10% NaOH (aq), and the aqueous mixture was extracted with hot CHCl_3_ (10 mL x 19). The organic extracts were combined, dried over K_2_CO_3_, and evaporated to give a colorless oil, which was used directly in the next step.

To a stirred solution of the above alcohol in CH_2_Cl_2_ (10 mL) was added 1,1,1-tris(acetyloxy)-1,1-dihydro-1,2-benzoiodoxol-3(1*H*)-one (Dess-Martin reagent, 97%, 283 mg, 0.65 mmol) at room temperature, and the reaction mixture was stirred at the same temperature for 3 h. The reaction was quenched with 10% Na_2_S_2_O_3_ in satd. NaHCO_3_ (aq), and the organic layer was separated. The aqueous layer was extracted with CH_2_Cl_2_ (10 mL x 2), and the organic layer and extracts were combined, dried over K_2_CO_3_, and evaporated to give a pale yellow oil, which was used directly in the next step.

To a stirred suspension of ICH_2_P^+^Ph_3_I^-^ (330 mg, 0.62 mmol) in THF (5 mL) was added a solution of NaHMDS (1M in THF, 0.6 mL, 0.6 mmol) at room temperature, and the reaction mixture was stirred at room temperature for 1 min. To the reaction mixture were added HMPA (0.2 mL) and then dropwise a solution of the above aldehyde in THF (2 mL) via a double-tipped stainless steel needle at –78 °C. The reaction mixture was stirred at –78 °C~rt for 2 h. and quenched with satd. NaHCO_3_ (aq). The aqueous mixture was extracted with Et_2_O (15 mL x 4), and the organic extracts were combined, dried over K_2_CO_3_, and evaporated to give a pale yellow oil, which was chromatographed on silica gel (15 g, hexane : acetone = 90 : 1~20 : 1) to afford **15** (91 mg, 60%) as a pale yellow oil.

IR (neat) 2925, 2873, 2780, 2705, 1457, 1263, 1134 cm^-1^; ^1^H NMR (500 MHz) δ 0.88 (3H, d, *J* = 6.8 Hz), 0.97 (1H, qd-like, *J* = 11.9, 4.3 Hz), 1.27-1.64 (5H, br m), 1.68-1.79 (3H, br m), 1.95 (1H, br), 2.11 (2H, br), 2.31 (1H, br), 2.48-2.51 (1H, m), 3.30 (1H, br), 6.26-6.31 (2H, m); ^13^C NMR (75 MHz) δ 18.85 (q), 20.36 (t), 28.90 (t), 31.21 (t), 33.43 (t), 36.16 (d), 39.77 (t), 51.87 (t), 62.23 (d), 71.39 (d), 84.20 (d), 138.06 (d); MS: 305 (M^+^); HRMS: Calcd for C_12_H_20_IN 305.0640; Found 305.0647; [α]_D_^26^ –80.1 (*c* 1.95, CHCl_3_).

**(5*S*,8*R*,9*S*)-(-)-8-Methyl-5-[5-(trimethylsilyl)pent-2-en-4-ynyl]octahydroindolizine (16)**

To a stirred suspension of CuI (18 mg, 0.093 mmol) and Pd(Ph_3_P)_4_ (36 mg, 0.031 mmol) in THF (2 mL) was added *i*-Pr_2_NH (0.26 mL, 1.87 mmol) at room temperature. To the resulting solution was added dropwise a solution of **15** (95 mg, 0.31 mmol) and TMS-acetylene (0.1 mL, 0.93 mmol) in THF (3 mL) via a double-tipped stainless steel needle. The reaction mixture was stirred at room temperature for 20 h., and then quenched with satd. NaHCO_3_ (aq). The aqueous mixture was extracted with Et_2_O (10 mL x 4), and the organic extracts were combined, dried over K_2_CO_3_, and evaporated to give pale yellow oil, which was chromatographed on silica gel (15 g, hexane : acetone = 100 : 1~20 : 1) to afford **16** (80 mg, 93%) as pale yellow oil.

IR (neat) 2958, 2780, 2149, 1250, 843 cm^-1^; ^1^H NMR (500 MHz) δ 0.19 (9H, s), 0.87 (3H, d, *J* = 6.4 Hz), 0.95 (1H, qm, *J* = 12.8 Hz), 1.31-1.53 (4H, br m), 1.66-1.82 (4H, br m), 1.90-1.96 (1H, m), 2.01-2.05 (2H, m), 2.38-2.44 (1H, m), 2.66-2.71 (1H, m), 3.32 (1H, td-like, *J* = 8.5, 1.7 Hz), 5.56 (1H, d-like, *J* = 9.4 Hz), 6.02 (1H, dt-like, *J* = 9.4, 7.3 Hz); ^13^C NMR (75 MHz) δ 9.89 (q), 18.92 (q), 20.42 (t), 29.08 (t), 31.58 (t), 33.59 (t), 35.66 (t), 36.49 (d), 52.00 (t), 63.18 (d), 71.39 (d), 98.89 (s), 101.93 (s), 110.61 (d), 142.04 (d); MS: 275 (M^+^); HRMS: Calcd for C_17_H_29_NSi 275.2068; Found 275.2077; [α]_D_^26^ –73.1 (*c* 3.41, CHCl_3_).

**(5*S*,8*R*,9*S*)-(-)-8-Methyl-5-(pent-2-en-4-ynyl)octahydroindolizine (203A)**

To a stirred solution of **16** (60 mg, 0.22 mmol) in MeOH (1 mL) was added K_2_CO_3_ (60 mg, 0.44 mmol), and the reaction mixture was stirred at room temperature for 1 h. The reaction mixture was diluted with Et_2_O, and the insoluble material was re3moved by filtration. The filtrate was evaporated to give indolizidine **203A** (40 mg, 91%) as a colorless oil.

IR (neat) 3311, 2953, 2925, 2872, 2849, 2780, 2702, 2359, 1457, 1162, 1135 cm^-1^; ^1^H NMR (500 MHz) δ 0.86 (3H, d, *J* = 6.4 Hz), 0.91-0.99 (1H, m), 1.30-1.49 (3H, br m), 1.51-1.57 (1H, m), 1.60-1.78 (4H, br m), 1.88-1.94 (1H, m), 1.99-2.05 (2H, q-like, *J* = 9.0 Hz), 2.46 (1H, dt-like, *J* = 14.5, 8.1 Hz), 2.62-2.68 (1H, m), 3.07 (1H, br s), 3.28 (1H, t-like, *J* = 8.5 Hz), 5.51 (1H, d-like, *J* = 10.9 Hz), 6.07 (1H, dt-like, *J* = 10.9, 7.3 Hz); ^13^C NMR (75 MHz) δ 18.92 (q), 20.45 (t), 29.08 (t), 31.49 (t), 33.59 (t), 35.73 (t), 36.50 (d), 51.95 (t), 62.79 (d), 71.31 (d), 80.45 (s), 81.58 (d), 109.47 (d), 142.79 (d); MS: 203 (M^+^); HRMS: Calcd for C_14_H_21_N 203.1673; Found 203.1659; [α]_D_^26^ –94.5 (*c* 2.00, CHCl_3_), Lit.^15^ [α]_D_ –23.3 (*c* 0.30, CHCl_3_).

**(5*S*,8*R*,9*S*)-5-[4-(*tert*-Butyldiphenylsilyloxy)but-1-enyl]-8-methyloctahydroindolizine (17)**

To a stirred solution of **13** (470 mg, 2.57 mmol) in THF (30 mL) was added LiAlH_4_ (292 mg, 7.70 mmol) at 0 °C, and the resulting suspension was refluxed for 15 h. After cooling, the reaction was quenched with 10% NaOH (aq), and the aqueous mixture was extracted with hot CHCl_3_ (15 mL x 10). The organic extracts were combined, dried over K_2_CO_3_, and evaporated to give a colorless oil, which was used directly in the next step.

To a stirred solution of (COCl)_2_ (0.34 mL, 3.86 mmol) in CH_2_Cl_2_ (6 mL) was added DMSO (0.55 mL, 7.71 mmol) at –78 °C, and the reaction mixture was stirred at –78 °C for 5 min. To the resulting mixture was added dropwise a solution of the above alcohol in CH_2_Cl_2_ (9 mL) via a double-tipped stainless steel needle at –78 °C, and the reaction mixture was stirred at the same temperature for 30 min. Triethylamine (1.6 mL, 11.57 mmol) was added to the reaction mixture, and the reaction mixture was warmed to 0 °C for 1 h. The reaction was quenched with satd. NaHCO_3_ (aq), and the organic layer was separated. The aqueous mixture was extracted with CH_2_Cl_2_ (15 mL x 2), and the organic layer and extracts were combined, dried over K_2_CO_3_, and evaporated to give a pale yellow oil, which was used directly in the next step.

To a stirred suspension of TBDPSO(CH_2_)_3_P^+^Ph_3_Br^-^ (5.4 g, 8.45 mmol) in THF (30 mL) was added a solution of *n*-BuLi (1.6 M in hexane, 5 mL, 7.97 mmol) at 0 °C, and the resulting orange solution was stirred at 0 °C for 10 min. To the reaction mixture was added dropwise a solution of the above aldehyde in THF (9 mL) via a double-tipped stainless steel needle at 0 °C. After stirring the reaction mixture for 18 h, the reaction was quenched with H_2_O. The aqueous mixture was extracted with Et_2_O (30 mL x 3), and the organic extracts were combined, dried over K_2_CO_3_, and evaporated to give a pale yellow oil, which was chromatographed on silica gel (50 g, hexane : acetone = 100 : 1~20 : 1) to afford **17** (949 mg, 83%) as a pale yellow oil.

^1^H NMR (500 MHz) δ 0.90 (3H, d, *J* = 6.4 Hz), 0.99-1.04 (1H, m), 1.07 (9H, s), 1.36-1.61 (6H, br m), 1.70-1.74 (2H, m), 1.84 (1H, q-like, *J* = 8.9 Hz), 1.93-1.97 (1H, m), 2.36-2.41 (2H, m), 2.70-2.74 (1H, m), 3.10 (1H, t-like, *J* = 8.5 Hz), 3.68 (2H, t, *J* = 6.9 Hz), 5.41-5.48 (2H, m), 7.38-7.46 (6H, m), 7.69-7.71 (4H, m).

**(5*S*,8*R*,9*S*)-(-)-5-(4-hydroxybutyl)-8-methyloctahydroindolizine (18)**

To a stirred solution of **17** (121 mg, 0.27 mmol) in EtOAc (5 mL) was added 10% Pd/C (50 mg), and the resulting suspension was hydrogenated at 1 atm under hydrogen atmosphere for 37 h. The catalyst was removed by filtration and the filtrate was evaporated to give a pale yellow oil. To the solution of this oil in THF (8 mL) was added a solution of TBAF (1 M in THF, 0.7 mL, 0.68 mmol), and the reaction mixture was stirred at room temperature for 14 h. The solvent was evaporated and the residue was chromatographed on silica gel (10 g, hexane : acetone = 30 : 1~1 : 1) to afford **18** (45 mg, 79%) as a pale yellow oil.

IR (neat) 3309, 2928, 2870, 2785 cm^-1^; ^1^H NMR (500 MHz) δ 0.87 (3H, d, *J* = 6.8 Hz), 0.95 (1H, qd, *J* = 12.8, 4.3 Hz), 1.26-1.77 (15H, br m), 1.91-1.97 (2H, br), 2.00 (1H, q-like, *J* = 9.0 Hz), 3.28 (1H, t-like, *J* = 8.6 Hz), 3.65 (2H, t-like, *J* = 6.8 Hz); ^13^C NMR (75 MHz) δ 18.95 (q), 20.39 (t), 22.09 (t), 29.01 (t), 31.07 (t), 33.17 (t), 33.64 (t), 34.30 (t), 36.39 (d), 51.79 (t), 62.78 (t), 63.49 (d), 71.39 (d); MS: 211 (M^+^); HRMS: Calcd for C_13_H_25_NO 211.1935; Found 211.1944; [α]_D_^26^ –112.2 (*c* 1.98, CHCl_3_).

**(5*S*,8*R*,9*S*)-(-)-8-Methyl-5-(pent-4-ynyl)octahydroindolizine (205A)**

To a stirred solution of (COCl)_2_ (0.1 mL, 1.14 mmol) in CH_2_Cl_2_ (1 mL) was added DMSO (0.17 mL, 2.40 mmol) at –78 °C, and the reaction mixture was stirred at –78 °C for 5 min. To the resulting mixture was added dropwise a solution of **15** (100 mg, 0.47 mmol) in CH_2_Cl_2_ (2 mL) via a double-tipped stainless steel needle at –78 °C, and the reaction mixture was stirred at –78 °C for 30 min. Triethylamine (0.5 mL, 3.61 mmol) was added to the reaction mixture, and the reaction mixture was warmed to 0 °C for 1 h. The reaction was quenched with satd. NaHCO_3_ (aq), and the aqueous mixture was extracted with CH_2_Cl_2_ (10 mL x 3). The organic layers were combined, dried over K_2_CO_3_, and evaporated to give a pale yellow oil, which was used directly in the next step.

To a stirred solution of (MeO)_2_P(O)CHN_2_ (248 mg, 1.66 mmol) in THF (2 mL) was added *t*-BuOK (186 mg, 1.66 mmol) at –78 °C, and the resulting solution was stirred at the same temperature for 5 min. To the solution was added dropwise a solution of the above aldehyde in THF (3 mL) via a double-tipped stainless steel needle at –78 °C, and the reaction mixture was stirred at-78 °C ~ room temperature for 2 h. The reaction was quenched with satd. NaHCO_3_ (aq), and the aqueous mixture was extracted with CH_2_Cl_2_ (10 mL x 4). The organic extracts were combined, dried, and evaporated to give a pale yellow oil, which was chromatographed on silica gel (15 g, hexane : acetone = 30 : 1~20 : 1) to afford **205A** (**33**, 60 mg, 62%) as a pale yellow oil.

IR (neat) 3311, 2951, 2871, 2778, 2117, 1457 cm^-1^; ^1^H NMR (500 MHz) δ 0.83 (3H, d, *J* = 6.9 Hz), 0.92 (1H, qm, *J* = 11.1 Hz), 1.18-1.33 (3H, m), 1.34-1.50 (4H, m), 1.58-1.65 (2H, m), 1.67-1.76 (4H, m), 1.84-1.98 (4H, m), 2.16 (2H, t-like, *J* = 6.5 Hz), 3.24 (1H, t, *J* = 9.0 Hz); ^13^C NMR (75 MHz) δ 18.77 (t), 18.88 (q), 20.36 (t), 24.77 (t), 29.04 (t), 31.15 (t), 33.59 (t), 33.67 (t), 36.47 (d), 51.76 (t), 62.86 (d), 68.30 (d), 71.22 (d), 84.25 (s); MS: 205 (M^+^); [α]_D_^26^ –80.6 (*c* 3.0, MeOH), Lit.^20^ [α]_D_^24^ -81.7 (c 0.36, MeOH).

**(5*S*,8*R*,9*S*)-5-[4-(*tert*-Butyldiphenylsilyloxy)but-1-enyl]-8-ethyloctahydroindolizine (19)**

To a stirred solution of **2** (216 mg, 1.10 mmol) in THF (10 mL) was added LiAlH_4_ (83 mg, 2.19 mmol) at 0 °C, and the resulting suspension was refluxed for 13 h. After cooling, the reaction was quenched with 10% NaOH (aq), and the aqueous mixture was extracted with hot CHCl_3_ (10 mL x 10). The organic extracts were combined, dried over K_2_CO_3_, and evaporated to give a colorless oil, which was used directly in the next step.

To a stirred solution of (COCl)_2_ (0.14 mL, 1.64 mmol) in CH_2_Cl_2_ (2 mL) was added DMSO (0.23 mL, 3.29 mmol) at –78 °C, and the reaction mixture was stirred at –78 °C for 5 min. To the resulting mixture was added dropwise a solution of the above alcohol in CH_2_Cl_2_ (3 mL) via a double-tipped stainless steel needle at –78 °C, and the reaction mixture was stirred at the same temperature for 30 min. Triethylamine (0.68 mL, 4.93 mmol) was added to the reaction mixture, and the reaction mixture was warmed to 0 °C for 1 h. The reaction was quenched with satd. NaHCO_3_ (aq), and the organic layer was separated. The aqueous mixture was extracted with CH_2_Cl_2_ (15 mL x 2), and the organic layer and extracts were combined, dried over K_2_CO_3_, and evaporated to give pale yellow oil, which was used directly in the next step.

To a stirred suspension of TBDPSO(CH_2_)_3_P^+^Ph_3_Br^-^ (2.8 g, 4.38 mmol) in THF (15 mL) was added a solution of *n*-BuLi (1.6 M in hexane, 2.5 mL, 3.95 mmol) at 0 °C, and the resulting orange solution was stirred at 0 °C for 10 min. To the reaction mixture was added dropwise a solution of the above aldehyde in THF (6 mL) via a double-tipped stainless steel needle at 0 °C. After stirring the reaction mixture for 16 h, the reaction was quenched with H_2_O. The aqueous mixture was extracted with Et_2_O (20 mL x 3), and the organic extracts were combined, dried over K_2_CO_3_, and evaporated to give a pale yellow oil, which was chromatographed on silica gel (20 g, hexane : acetone = 100 : 1~20 : 1) to afford **19** (362 mg, 72%) as a pale yellow oil.

^1^H NMR (500 MHz) δ 0.90 (1H, m), 0.91 (3H, t, *J* = 7.3 Hz), 1.06 (9H, s), 1.11-1.60 (9H, br m), 1.64-1.95 (3H, br m), 2.38 (2H, q-like, *J* = 6.7 Hz), 2.71 (1H, br), 3.09 (1H, br), 3.67 (2H, t, *J* = 6.8 Hz), 5.44 (2H, br), 7.38-7.46 (6H, m), 7.67-7.74 (4H, m).

**(5*S*,8*R*,9*S*)-(-)-5-(4-hydroxybutyl)-8-ethyloctahydroindolizine (20)**

To a stirred solution of the olefin (19, 362 mg, 0.79 mmol) in EtOAc (20 mL) was added 10% Pd/C (250 mg), and the resulting suspension was hydrogenated at 1 atm under hydrogen atmosphere for 40 h. The catalyst was removed by filtration and the filtrate was evaporated to a give pale yellow oil. To the solution of this oil in THF (15 mL) was added a solution of TBAF (1 M in THF, 2.4 mL, 2.36 mmol), and the reaction mixture was stirred at room temperature for 18 h. The solvent was evaporated and the residue was chromatographed on silica gel (15 g, hexane : acetone = 30 : 1~1 : 1) to afford **20** (143 mg, 81%) as a pale yellow oil.

IR (neat) 3334, 2934, 2862, 2785, 1459 cm^-1^; ^1^H NMR (500 MHz) δ 0.82-0.90 (1H, m), 0.87 (3H, t, *J* = 7.4 Hz), 1.00-1.09 (1H, m), 1.12-1.23 (2H, m), 1.26-1.36 (2H, m), 1.44-1.82 (13H, br m), 1.84-1.98 (4H, m), 2.20 (1H, br), 3.25 (1H, t, *J* = 6.8 Hz), 3.62 (2H, t-like, *J* = 6.0 Hz); ^13^C NMR (75 MHz) δ 11.12 (q), 20.44 (t), 22.09 (t), 25.95 (t), 29.06 (t), 29.80 (t), 30.97 (t), 33.17 (t), 34.34 (t), 42.86 (d), 51.84 (t), 62.58 (t), 63.49 (d), 69.96 (d); MS: 225 (M^+^); HRMS: Calcd for C_14_H_27_NO 225.2091; Found 225.2097; [α]_D_^26^ –117.98 (*c* 4.44, CHCl_3_).

**(5*S*,8*R*,9*S*)-(-)-8-Ethyl-5-(pent-4-ynyl)octahydroindolizine (219F)**

To a stirred solution of (COCl)_2_ (0.1 mL, 1.14 mmol) in CH_2_Cl_2_ (1 mL) was added DMSO (0.17 mL, 2.40 mmol) at –78 °C, and the reaction mixture was stirred at –78 °C for 5 min. To the resulting mixture was added dropwise a solution of **20** (80 mg, 0.36 mmol) in CH_2_Cl_2_ (2 mL) via a double-tipped stainless steel needle at –78 °C, and the reaction mixture was stirred at –78 °C for 30 min. Triethylamine (0.5 mL, 3.61 mmol) was added to the reaction mixture, and the reaction mixture was warmed to 0 °C for 1 h. The reaction was quenched with satd. NaHCO_3_ (aq), and the aqueous mixture was extracted with CH_2_Cl_2_ (10 mL x 3). The organic layers were combined, dried over K_2_CO_3_, and evaporated to give a pale yellow oil, which was used directly in the next step.

To a stirred solution of (MeO)_2_P(O)CHN_2_ (190 mg, 1.26 mmol) in THF (2 mL) was added *t*-BuOK (142 mg, 1.26 mmol) at –78 °C, and the resulting solution was stirred at the same temperature for 5 min. To the solution was added dropwise a solution of the above aldehyde in THF (3 mL) via a double-tipped stainless steel needle at –78 °C, and the reaction mixture was stirred at-78 °C ~ room temperature for 2 h. The reaction was quenched with satd. NaHCO_3_ (aq), and the aqueous mixture was extracted with CH_2_Cl_2_ (10 mL x 4). The organic extracts were combined, dried, and evaporated to give a pale yellow oil, which was chromatographed on silica gel (15 g, hexane : acetone = 30 : 1~20 : 1) to afford **219F** (50 mg, 64%) as a pale yellow oil.

IR (neat) 3312, 2932, 2864, 2778, 1457 cm^-1^; ^1^H NMR (500 MHz) δ 0.87 (3H, t, *J* = 7.3 Hz), 0.82-0.92 (1H, m), 1.00-1.09 (1H, m), 1.12-1.26 (2H, br m), 1.38-1.51 (4H, m), 1.54-1.66 (3H, m), 1.71-1.80 (3H, m), 1.85-1.99 (5H, br m), 2.18 (2H, td-like, *J* = 7.3, 3.8 Hz), 3.26 (1H, t-like, *J* = 7.7 Hz); ^13^C NMR (75 MHz) δ 11.15 (q), 18.82 (t), 20.47 (t), 24.82 (t), 25.97 (t), 29.09 (t), 29.79 (t), 31.03 (t), 33.66 (t), 42.94 (d), 51.83 (t), 62.97 (d), 68.34 (d), 69.95 (d), 84.33 (s); MS: 219 (M^+^); HRMS: Calcd for C_15_H_25_N 219.1986; Found 219.1993; [α]_D_^26^ –102.48 (*c* 2.05, CHCl_3_).
